# Measuring quality in diabetes care: an expert-based statistical approach

**DOI:** 10.1186/2193-1801-2-226

**Published:** 2013-05-16

**Authors:** Dimitris Bertsimas, David Czerwinski, Michael Kane

**Affiliations:** Sloan School of Management, Massachusetts Institute of Technology, E40-147 Cambridge, MA 02139 USA; Department of Marketing and Decision Sciences, San Jose State University, San Jose, CA 95192-0069 USA; MIT Medical, Building E23, 77 Massachusetts Avenue, Cambridge, MA 02139-4307 USA

**Keywords:** Quality of care, Claims data, Logistic regression, Diabetes

## Abstract

We present a methodology for using health insurance claims data to monitor quality of care. The method uses a statistical model trained on the quality ratings of a medical expert. In a pilot study, the expert rated the quality of care received over the course of two years by 101 diabetes patients. A logistic regression model accurately identified the quality of care for 86% of the patients. Because the model uses data derived from patients’ health insurance claims it can be used to monitor the care being received by a large patient population. One important use of the model is to identify potential candidates for case management, especially patients with complicated medical histories.

## Background

Researchers have demonstrated in recent years that many patients in the United States do not receive high-quality health care ( Schuster et al. 1998 ; Corrigan 2001 ). In this paper, we address the problem of identifying, in an automated fashion, diabetes patients who may be receiving poor care so that interventions can be arranged to improve their care. We measure quality of care with an expert-trained statistical model using variables derived from medical insurance claims data.

We focus on patients with diabetes for several reasons. First, diabetes is a widespread, costly disease. Over 25 million Americans are diabetic – about 8% of the US population – and the annual cost of diabetes is estimated at $174 billion ( Centers for Disease Control and Prevention 2011 ). Second, there are well established guidelines for its treatment, and third, limiting the study to one disease minimizes variations in care from patient to patient that aren’t related to quality.

We are interested in identifying individual patients with poor care in “real-time” so that interventions can be arranged to improve their care. This intervention-oriented patient-centered view is somewhat distinct from previous studies of quality of care. Existing studies have measured quality in order to assess the US healthcare system ( Schuster et al. 1998 ; Corrigan 2001 ; Berwick 2002 ; Davis et al. 2007 ; Agency for Healthcare Research and Quality 2006 ; Schoenbaum et al. 2007 ; Asch et al. 2004 ; Leatherman and McCarthy 2002 ; Hayward 2007 ); to study health plans ( Wholey et al. 2003a Wholey et al. 2003b ); to identify practices and factors that improve quality ( Heisler and Wagner 2004 ; Solberg et al. 2008 ; Hanchate et al. 2010 ); to provide rankings of doctors and healthcare facilities ( United States Department Of Health and Human Services 2012 ; Hofer et al. 1999 ; Gandhi et al. 2002 ; Li et al. 2007 ; Shwartz et al. 2009 ); and to rate providers for “pay-for-performance” type reimbursement ( Pham et al. 2007 ; Scholle et al. 2009 ; Romano and Mutter 2004 ).

We want to emphasize that the goal of identifying poor care is not to assign blame for it. Poor quality care can be caused by the physician or the patient (or perhaps the combination of a particular patient with a particular physician) or external factors beyond either’s control. The purpose of the model is to identify patients receiving poor care so that case management can be arranged. The case management might very well focus on improving the patient’s compliance with care.

To measure quality, we use a statistical model trained on a set of patients whose care was assessed by a physician. The variables in the model are derived from the patients’ insurance claims data. We use claims data because in practice they are the only electronically available, timely source of information documenting the care a patient has received. With them, the care received by a large population of patients can be monitored on an ongoing basis. Other methods of measuring quality, such as reviewing paper medical records, may be more thorough but they do not scale to large patient populations because of the manual labor involved.

That is not to say that claims data are without drawbacks. They lack clinical details such as symptoms, test results, and severity of disease. They reflect little about the patient’s quality of life. Though overall the coding of diagnoses and procedures in claims data are accurate, they can sometimes be vague ( Kashner 1998 ). When there are multiple diagnoses during a single visit some may not be captured. Minor non-monetized procedures – such as counseling a patient to stop smoking – are usually not recorded.

Our statistical models measure the quality of the process of care ( Brook et al. 2000 ; Brook and Cleary 1996 ). In trying to improve the care for a particular patient, structural aspects of care are less relevant since they are fixed over the short term. Ideally, we would measure outcomes of care but outcomes are difficult to infer from claims data since lab results, symptoms, etc. are not captured ( Wennberg et al. 1987 ). For example, an insurance claim may record that a diabetes patient had a glycated hemoglobin test, but it will not record the results of the test. Whether the patient’s glucose level is improving or not cannot be determined. Furthermore, because we have data only over a two-year period, long-term outcomes cannot be measured. Though we didn’t instruct the physician rating the quality of care to look specifically at process of care, that is what *de facto* was available to him.

Of course, process measures of quality of care for diabetes patients exist in the form of the guidelines of the American Diabetes Association ( 2007 ). These guidelines have been developed based on the best available evidence and, where conclusive evidence is still lacking, consensus of expert opinion. Yet, many aspects of a patient’s care are beyond the purview of the guidelines. Also, using clinical guidelines to measure quality of care for patients with multiple diseases can be problematic because guidelines focus on the optimal treatment of a single condition ( Kerr et al. 2001 ). For an individual with several coexistent disorders the treatment demands for one disease may conflict with recommendations for others. Finally, intangible aspects of care might be difficult to capture in written guidelines.

By having a physician review the claims data for our study set, we obtain a holistic view of the patient’s care. The physician can consider not only the care they received for their diabetes, but also for comorbidities. He can examine the patient’s overall course of care, including routine preventive care. Nevertheless, the diabetes treatment guidelines are relevant and below we discuss how compliance with the guidelines correlated with the physician’s assessment of care.

Though we have built a statistical model to identify poor quality care, we do not claim that the model *defines* poor care. Identifying poor care is not equivalent to defining it. For example, consider the statistical models used by credit card companies to identify fraudulent patterns of transactions. The use of a credit card in rapid succession at gas stations may be a red-flag that the card has been stolen. But that is not to say that it is wrong for a person to use their own credit card in rapid succession at gas stations. It is simply a fact that such behavior is correlated with fraud. In the same way, if our statistical models incorporate the use of narcotics as a red-flag for poor care this does not mean that all uses of narcotics are inappropriate. It simply means that there is a correlation between the use of narcotics and poor care. An advantage of our approach is that it doesn’t rely on an explicit definition of quality.

## Methods

From a large health insurance claims database, we randomly selected 101 diabetes patients aged 35–55 with healthcare costs between $10,000 and $20,000 over the two-year study period (September 1, 2003 to August 31, 2005). A patient was considered diabetic if they had either two outpatient diagnoses of diabetes or one inpatient diagnosis of diabetes over the two-year period. The lower bound on the cost ensured that each patient had enough claims data so that the reviewer could make an assessment of the quality of care they received. Likewise, the upper bound ensured that no patient’s claims record was so lengthy that it would be impractical to review.

We attempted to oversample patients who might have received poor care to ensure their adequate representation in the sample. Of course, without a measure of the quality of care at the outset, we couldn’t do this exactly. As an approximation, we scored the patients based on the presence of hemoglobin HbA1c tests, lipid profiles, and eye exams in their claims data ( Weiner et al. 1995 ). Scores could range from zero (none of the above procedures was performed) to three (all three procedures were performed). We then drew a stratified random sample by score, oversampling the lower scores.

The claims data consist of all insurance-based health care utilization for the patients in the study and comprise medical services and pharmaceutical prescriptions. Claims for medical services record the date of service, the provider, diagnoses, procedures performed, and the amount paid. Claims for prescription drugs record the date the prescription was filled, the prescribing physician, the drug, the number of days of supply, and the amount paid.

One of us, physician Dr. K, reviewed the claims records for each of the 101 patients and scored the quality of care they received. He rated the care on a three-point scale: poor, average, or good. He also rated his confidence in his assessment on a two-point scale: confident or not confident. In addition, he wrote a one-paragraph summary for each patient describing their condition and the care they received and noting aspects of their care that influenced his rating. These narratives were used later in developing variables for the statistical model.

Dr. K reviewed and rated 30 additional patients (not used to develop the model) which were used to validate the model. A second physician, Dr. L, also rated the 30 patients in the validation set independently of Dr. K. Having a second physician rate the patients allowed us to assess the extent to which the models reflected beliefs about quality specific to Dr. K. The contrast between the backgrounds and experience of the two doctors is marked. Dr. K was trained in the United States and has over 30 years of experience, whereas Dr. L was trained abroad and had recently completed his residency.

### Model development and evaluation

Our dependent variable is quality of care. Since we are mainly concerned with identifying poor quality care, we combined patients who received “average” or “good” care into a single group which we will refer to as the good care group. A value of 1 for the quality of care variable was used to indicate good quality care, a value of 0 for poor. Incorporating information about the physician’s confidence did not lead to more accurate models nor did disaggregating the good care group into average care and good care.

Though the number of independent variables that we explored is large, we provide a brief overview of them here. The full list of variables with their definitions can be found in the Appendix. The variables may be categorized into those related to diabetes treatment (e.g. the number of glycated hemoglobin tests performed), patient demographics (e.g. age and gender), healthcare utilization (e.g. total number of office visits), markers of good care (e.g. the performance of a mammogram for females), markers of poor care (e.g. the administration of a B12 injection), providers (e.g. number of different providers), claims (e.g. total number of claims), and prescriptions (e.g. number of prescriptions and changes in prescriptions).

All of the independent variables are computable directly from the patients’ claims data. Most of the variables capture general aspects of care but some are specifically inspired by the physician’s comments in his narratives. Yet we avoided defining variables that would only apply to one or two patients in the sample since no statistically meaningful statements could be made about such variables. For example, one patient was judged to have received poor care because she was treated over a long period with an antibiotic for a urinary tract infection but without regular gynecological exams. (When she finally did have a gynecological exam uterine cancer was discovered.) As this situation arose with only one patient, we would not be able to statistically assess the value of a quality indicator such as “on antibiotic for a urinary tract infection without gynecological exams.” We restricted our attention to variables that would apply more broadly.

We modeled the data using logistic regression. To assess a model’s accuracy we computed the percent of patients that it classified correctly (i.e. that matched the physician’s classification). Bootstrap resampling was used to estimate out-of-sample accuracy during model development due to the small number of observations. 500 bootstrap trials were used, adjusting for bias as discussed in Hastie et al. ( 2001 ). After model selection and fitting was complete we performed a true out-of-sample test on the 30 hold-out cases.

Because the majority of patients received good care, the simplest predictive model would be to blindly classify each patient’s care as good; 78% of patients would be accurately classified. This serves as a useful baseline against which to assess our model. Another natural baseline model is one using only variables based on the diabetes treatment guidelines. A logistic regression model based on only these variables also had an accuracy of 78%. Comparing the performance of our model to such a model reveals whether incorpating aspects of care beyond the guidelines is of value in identifying poor care.

We used a stepwise procedure to develop the model. We started with an empty model and continued to add variables until the bootstrap estimate of out-of-sample accuracy stopped improving.

## Results

### Data summary

Table 1 shows a summary of the physician’s quality-of-care ratings for the 101 patients. 78% of the patients received average or better care. The physician had high confidence in 76% of his assessments. Most of the cases of low confidence occurred in the “average care” group (*χ*^2^=10.6, *p* = 0.005).

**Table 1:** **Summary of the physician’s quality ratings and his confidence in them**

	Low confidence	High confidence
Low quality	5	17
Average quality	16	25
High quality	3	35

Here is an example descriptive paragraph written by the physician for a patient who received good care:

45 year old type 2 diabetic on metformin and glyburide. Also took lexapro and ambien regularly, and crestor. He carried a diagnosis of sarcoid for first part of the analysis period and was treated with prednisone for a while-that’s appropriate for sarcoid, even in a diabetic. He was also appropriately covered with fosamax initially. He seems to have changed PCP’s in mid cycle. Had first labs 2/04. Had only one ER visit for a forehead laceration. He had a stress test 5/16/05 for chest pain, followed by a catheterization on 5/27/05. Apparently nothing worrisome was found. Despite sarcoid diagnosis he had no chest X-rays or pulmonary function tests. Had one podiatry visit in June ’05. No home testing, no eye exams. Overall, given pulmonary and mental health comorbidities, care looks good with high confidence.

To determine how much variability there was in diabetes care among the patients in the sample we assessed the compliance of the patients’ care with three components of the diabetes treatment guidelines (eye exams, glycated hemoglobin tests, and lipid profiles).

36% of the patients in the sample had evidence of at least one eye exam. Since a patient may have had separate vision insurance, an eye exam may not have been recorded in the health insurance claims data even if one was performed. Therefore, this number should be treated as a lower bound on the percent of patients who had eye exams. 54% had evidence of a glycated hemoglobin test and 54% had evidence of a lipid profile. However, when laboratory tests such as these are performed in a hospital, the type of test is often not coded in the insurance claims data. 59 patients in the data set had instances of non-specified diabetes testing performed in hospitals. If we are generous and assume that when testing was done it was the *correct* tests (according to the guidelines), then the compliance would be 91% for hemoglobin tests and 92% for lipid profiles. Alternately, if we limit ourselves to the 36 patients all of whose lab work was performed outside of hospitals, 75% had hemoglobin tests and 78% had lipid profiles. The correlation between the performance of hemoglobin tests and the performance of lipid profiles was 0.46.

Table 2 shows the compliance with each component of the diabetes treatment guidelines for the poor, average, and good care groups. For example, of the patients whose care was rated poor, 26% received eye exams whereas 50% of the patients whose care was rated good received eye exams. For eye exams and hemoglobin tests, compliance tends to increase as the physician’s rating of quality increases. The differences are not statistically significant however (p= 0.95).

**Table 2:** **Percent of patients receiving eye exams, glycated hemoglobin tests, and lipid profiles by quality rating**

	Poor	Average	Good
Percent receiving eye exams	25	34	43
Percent receiving hemoglobin tests	40	53	62
Percent receiving lipid profiles	50	55	54

### Logistic regression model

The logistic regression model incorporates the variables StartedOnCombination, HemoglobinTest, and AcuteDrugGapSmall. StartedOnCombination is an indicator variable with a value of 1 indicating that the patient’s drug therapy for diabetes started with a combination of drugs, rather than a single drug. HemoglobinTest is a count of the number of glycated hemoglobin tests the patient had. AcuteDrugGapSmall is the fraction of refills for acute drugs that followed the exhaustion of the previous prescription by a gap of between 1 and 30 days.

The regression equation is:

In the model, polypharmacy (StartedOnCombination) is penalized through its negative coeffient. Glycated hemoglobin tests are rewarded, which is fitting as they are recommended by diabetes treatment guidelines. And finally, the variable AcuteDrugGapSmall has a negative coefficient. A high value for this variable indicates the repeated use of an acute drug. Such repeated use may indicate that the physician’s diagnosis or choice of drug is incorrect.

The model has an in-sample accuracy of 86% and an estimated out-of-sample accuracy of 84%. The model correctly identified 12 of the 22 cases of poor care for a sensitivity of 54%. Only four patients out of the 79 who received good care were misclassified, for a specificity of 95%. The agreement between Dr. K and the model is summarized in Table 3.

**Table 3:** **Dr. K’s classification compared with the model’s**

	Model	
Dr. K	Poor	Good
Poor	12	10
Good	4	75

**Figure 1:**
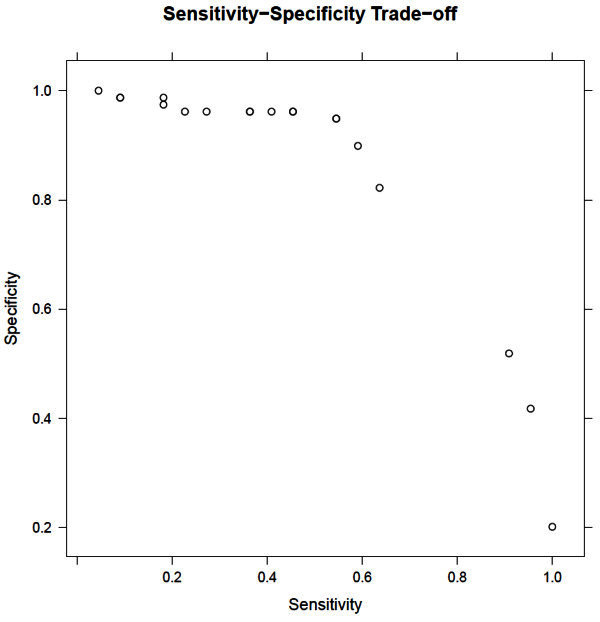
**Trade-offs between sensitivity and specificity obtainable using the logistic regression model.**

The cutoff value used in the logistic regression model can be used to adjust the trade-off between sensitivity and specificity. The accuracy of 86% was obtained by setting the cutoff value between poor care and good care at the level which maximizes overall accuracy. Figure 1 shows the range of sensitivity and specificity that can be obtained by our model by varying the cutoff. A higher sensitivity will result in a higher percentage of poor care cases being correctly identified. Though in practice, maintaining a high specificity may be more important.

### Out-of-sample validation

Because the patient mix is different for the hold-out sample of 30 than for the original training data, the relevant baseline accuracy for comparison is not 78% but 63%. (Direct comparison to the estimated out-of-sample accuracy rate therefore isn’t meaningful, though comparisons of sensitivity and specificity are.) When applied to the hold-out sample, our model has an accuracy of 80%, 17 percentage points above the baseline. The model has an out-of-sample sensitivity of 54% and specificity of 95%. The out-of-sample agreement between Dr. K and the model is summarized in Table 4.

**Table 4:** **Out-of-sample comparison of Dr. K’s classifications with the model**

	Model	
Dr. K	Poor	Good
Poor	6	5
Good	1	18

Table 5 shows the level of agreement between the two doctors’ ratings on the 30 out-of-sample cases. The doctors were in complete agreement on 12 of the 30 patients. On an additional 14 their ratings differed by a single level (e.g. a case rated as good by one doctor was rated average by the other). In only four cases was there a complete divergence of ratings, with one doctor rating the care as good and the other doctor rating it as poor. In three of these four cases, Dr. K was the one who rated the care poor and in all three cases Dr. K’s comments indicated that he felt the patient was on an inappropriate combination of drugs. Dr. L did not make comments about drug combinations for any of the 30 patients.

**Table 5:** **A comparison of the two physicians’ ratings**

	Dr. L		
Dr. K	Poor	Average	Good
Poor	4	4	3
Average	6	5	2
Good	1	2	3

Here are sample paragraphs written by the two physicians for one of the patients who both doctors felt received good care with high confidence. We begin with Dr. L’s assessment:

This patient was closely monitored for blood glucose, had ophthalmology follow up, multiple urinalysis, on an ACE ARB. Treated with multiple oral agents. Seen for foot problems. Might have benefited from a platelet inhibitor, but otherwise high quality care.

And Dr. K’s assessment:

She is a Type 2 diabetic on several oral agents. She also had regular prescriptions for an ACE inhibitor and for diabetes testing supplies. Had regular prescriptions for nortriptyline (antidepressant) and lorazepam with no formal mental health care, but I saw no sign of excess care or other issues that would indicate active mental health problems. She had eye, gyn and podiatry care and a mammogram, and regular visits with her PCP. She had an ER visit for abdominal pain in July ’04 with a prompt follow up visit afterward with her PCP. There were no hospitalizations. Care was orderly and looks to be good care with high confidence.

Neither doctor tended to be more negative in their overall ratings than the other. Dr. K rated 11 patients’ care as poor, 13 as average, and six as good. Dr. L rated 11 patients’ care as poor, 11 as average, and eight as good.

The doctors also provided confidence scores for their ratings. Dr. K rated his confidence as high for 25 of the patients, Dr. L for 21 of the patients. Since Dr. K has more experience reviewing claims data, this difference is not surprising. The doctors were jointly confident in their ratings of 18 of the patients. Their quality ratings were in complete agreement on nine of these 18 patients, a rate marginally better than for the whole set of 30 patients. They still reached opposite conclusions in three cases.

Our model performs only modestly when compared with Dr. L’s ratings. It has an accuracy of 67% in matching Dr. L’s ratings, with a sensitivity of 36% and specificity of 84%.

## Comment

We have demonstrated that an expert-trained statistical model using health insurance claims data can discriminate between the quality of care patients receive. Furthermore, only a simple model is required to capture approximately half of the cases of poor care while maintaining a low false-positive rate.

Although an accuracy of 86% may not sound terribly high, in the context of case management even this level of accuracy can be quite beneficial. A key challenge in case management is identifying patients to intervene with, and patients receiving poor care are ideal candidates. Suppose a case management company had 1000 diabetes patients, half of whom were receiving poor care. If a case manager blindly selected patients from this pool, they would waste half of their time on patients already receiving good care. On the other hand, suppose our model was applied to the 1000 diabetes patients and only those identified as receiving poor care were passed on to the case manager. With a sensitivity of 54%, the model would correctly identify 270 poor-care patients as such. With its specificity of 95%, the model would incorrectly classify 25 patients receiving good care.

Thus, the model would identify a total of 295 patients, 270 of whom actually were receiving poor care. Working from this “enriched” pool of patients, the case manager would waste little time on patients receiving poor care. Of the 295 patients, 92% would be receiving poor care – a significant improvement over the 50% rate if the patients were chosen blindly.

The key to the success of the model is not its sensitivity, which at 54% is admittedly modest. The key is the modest sensitivity coupled with a very high specificity so that cases of good care are filtered out. The model will in fact miss many cases of poor care. But a successful case management strategy need not involve identifying all the cases of poor care, it must simply identify enough to keep the case managers efficiently engaged.

Though we have focused on the use of logistic regression to *classify* patients, in practice the fitted probabilities could be used directly as numeric quality scores. For instance, the scores could be used to prioritize patients for case management. Rather than treating patients in the poor-care group as homogeneous, case managers could begin with the patient whose quality score was the lowest. Next, the patient with the second lowest quality score, and so on. In this way, resources would be focused first on the patients who may be the likeliest to be receiving poor quality care.

During the exploratory phase of model development, one unexpected relationship was revealed. A pattern emerged of an inverse relationship between the *quantity* of care ond the *quality* of care. There could be several reasons for this. For example, the more interactions a patient has with the health-care system the more opportunities there are for a mistake or other error to occur. An alternative explanation could be that some patients require so much care because the care they are receiving *is* poor: that is, the care is not making them better and so they continue to seek more care.

One limitation of our pilot study is that it only included quality ratings from two physicians. The lack of strong agreement between the two physician’s ratings underscores the fact that in a production setting, our approach would be best served by a panel of reviewers. Since a panel can be costly to convene, in practice the model could also be improved over time using input from the case managers. If a case manager using the model disagrees with the rating for a particular patient, they could record their own rating. As such ratings accumulated in a database, the model could then be re-fit. This approach could be a cost-effective way to create much larger training data sets with minimal additional overhead. Note that the use of case managers might tilt the outcome being measured from “quality of care” to “potential for intervention,” which is not necessarily undesirable.

There are several characteristics of the patients in our study that may limit the generality of the model. The patients are all insured, and this is necessary because the method relies on electronic insurance claims data. The patients are between the ages of 35 and 55. The general approach would apply to patients outside this range, though the specific model may change. The patients have relatively high health care costs. It may be difficult to apply this method to patients below a certain cost threshold, since the sparsity of the claims data may not provide enough information on which to base a judgment of quality. Although high-cost patients are of the most interest to insurers implementing case management, a specific weakness of this methodology is its insensitivity to evaluating the quality of care received by low-cost patients. A final limitation of the study is that we have used a relatively small sample size. More data, coupled with a panel of physician reviers as discussed above, will lead to more accurate and more generalizable models.

## Appendix

### Full list of variables

The variables we developed for our models are listed below, grouped into the following categories: diabetes treatment, patient, utilization, ratios, markers of good care, markers of poor care, providers, claims, and prescriptions.

### Diabetes treatment

The following variables are based on the guidelines for the treatment of diabetes or otherwise related to diabetes care.

**EyeExam** The number of eye exams. Note that this variable is hampered by the fact that some people may have visits with eye doctors that are not covered by their insurance or are covered by a different insurance plan.

**HemoglobinTest** The number of glycated hemoglobin tests.

**LipidProfile** The number of lipid profiles.

**GenericLab** The number of times unspecified lab work was performed (generally at a hospital where the details of the lab work are not recorded as carefully as for outpatient lab work). Multiple tests performed on the same day are considered as one occurrence.

**AnyLab** The number of times any of the following lab work was performed: glycated hemoglobin tests, lipid profiles, or unspecified lab work.

**DiabetesLab** This variable is similar to AnyLab with two differences: the diagnosis recorded with the lab work must be diabetes, and a broader range of lab work is included. The included lab work is: glycated hemoglobin tests, lipid profiles, unspecified lab work, hemoglobin tests, metabolic panels, urine microalbumin tests, and serum creatinine test.

**GlucoseSupplies** The number of times glucose testing supplies were ordered.

**AceInhibitors** The number of prescriptions for ace inhibitors.

**AceInhibitorDays** The number of days for which the patient had ace inhibitors prescribed. This may be a more accurate measure than the number of prescriptions, since prescriptions can vary in length.

**ARBs** The number of prescriptions for angiotensin II receptor blockers.

### Patient

The following variables reflect demographic information about the patient or about the patient’s claims data.

**Age** the patient’s age.

**Female** 1 if the patient is female, 0 otherwise.

**Diabetic** 1 if the patient is diabetic. (After reviewing each patient’s claims data, in the physician’s opinion a handful of patients in the study were not actually diabetics but were included due to spurious coding.)

**DrugsMissing** An indicator variable which is 1 if all pharmacy claims for the patient were unavailable.

**DiseaseCount** The number of chronic diseases that the patient had.

**Anxiolytics** The number of prescriptions for anxiolytics.

**Antidepressants** The number of prescriptions for antidepressants.

**Pain** 1 if the patient had any coding for pain, 0 otherwise.

**MedianMonthlyCost** The patient’s median monthly health-care cost over the study period.

**CostDerivative** The slope of the patient’s monthly costs over the study period. This was calculated for each patient by fitting a linear regression of monthly cost versus time (indexed 1,…,24 for the 24 months in the study period) and extracting the coefficient for time.

**CostSecondDerivative** The second derivative of the patient’s monthly costs over the study period. This was calculated for each patient by fitting a linear regression of monthly cost versus time, including a quadratic term, and taking the coefficient for the quadratic term.

### Utilization

**InpatientDays** The number of days spent in the hospital.

**ERVisits** The number of visits to an emergency room.

**OfficeVisits** The number of visits coded as 99213 or 99214 and taking place in an office or outpatient hospital setting.

**InpatientPerOffice** The ratio of inpatient days to office visits.

**ERPerOffice** The ratio of emergency room visits to office visits.

**TotalVisits** The sum of the number of office visits, emergency room visits, and inpatient days.

**ERVisits.normalized** ERVisits divided by TotalVisits.

**InpatientDays.normalized** InpatientDays divided by TotalVisits.

**OfficeVisits.normalized** OfficeVisits divided by TotalVisits.

**ER.outpatient** When an emergency room visit occurs, the percent of time that the next visit is an outpatient visit.

**ER.inpatient** When an emergency room visit occurs, the percent of time that the next visit is an inpatient visit.

**ER.ER** When an emergency room visit occurs, the percent of time that the next visit is another emergency room visit.

**ER.Other** When an emergency room visit occurs, the percent of time that the next visit is any other type of visit than outpatient, inpatient, or emergency.

**DaysSinceLastERVisit** The number of days between the patient’s last emergency room visits and the end of the study period. For patients who didn’t have any emergency room visits this was set equal to the length of the study period.

**PhysicalTherapy** The number of days on which the patient had physical therapy performed. (When a patient has physical therapy it often lasts a large number of days and this may drive up some of the quantity of care measures).

**Chiropractic** The number of days in which the patient had chiropractic services performed. As with physical therapy, chiropractic is usually performed a large number of times and this may drive up some of the quantity of care measures.

### Ratios

**GenericLabsPerOffice** GenericLab divided by OfficeVisits

**CostDrugRatio** The patient’s median monthly cost divided by the average number of chronic drugs they were on.

**InpatientDrugRatio** The number of days the patients spent in the hospital divided by the average number of chronic drugs they were on.

**DiseaseVisitsRatio** The number of chronic diseases divided by the number of visits.

**DiseaseRegularityRatio** The number of chronic diseases divided by VisitRegularity (see below).

### Markers of good care

The following variables correspond to aspects of the patient’s care that are considered to be markers of good care.

**Mammogram** The number of mammograms.

**BinaryMammogram** 1 if the patient had at least one mammogram, 0 otherwise.

**VisitRegularity** The study period was broken up into three-month intervals and VisitRegularity is the fraction of those intervals in which there was at least one claim.

**OfficeVisitRegularity** The same as VisitRegularity except that only office visits are counted, not all claims.

**LongestOfficeGap** The longest gap, in days, between successive office visits.

### Markers of poor care

**Narcotics** The number of prescriptions for narcotics.

**NarcoticsDays** The number of days for which the patient had narcotics prescribed.

**B12** The number of prescriptions or injections of vitamin B12. (Over-the-counter use of vitamin B12 would not be included if the patient paid for out of their own pocket.)

**Polypharmacy** An indicator variable which is 1 if the patient’s pharmaceutical treatment for diabetes was initiated with a combination of drugs at once. By default, the indicator is set to 0 for patients who were already on diabetes drugs at the beginning of the study period.

### Providers

**ProviderCount** The number of providers that served the patient. Note that along with physicians, a hospital, lab, or a clinic can be counted as a provider, as can anesthesiologists, pathologists, etc.

**PrescriberCount** The number of doctors who prescribed drugs for the patient.

**DiabetesProviders** The number of providers who treated the patient’s diabetes. We include all providers who had a claim for which diabetes was listed as the diagnosis.

**PrescribersPerProvider** The number of prescribers divided by the number of providers.

### Claims

**MedicalClaims** The number of days on which the patient had a medical claim (i.e. all claims except prescriptions).

**ClaimLines** The number of medical claims. This differs from MedicalClaims in that multiple claims on the same date are each counted.

**ClaimsPerDate** A patient may have multiple claims on any given date. This is the total number of claims a patient had divided by the number of dates on which the patient had claims. This variable is an attempt to tease out the “complexity” of a visit – presumably the more claims that occurred on a date the more complex the encounter.

### Prescriptions

**DrugsStarted** The number of drugs started during the study period. (Any drug for which the first prescription occurs within the first 90 days of the study period is not included since it is likely that the patient was already on the drug and is renewing their prescription.)

**DrugsEnded** The number of drugs stopped during the time period. (Any drug for which the last prescription occurs within the last 90 days of the study period is not included because the patient may have continued on the drug after the study period ended.)

**DrugsAtBeginning** The number of drugs the patient is on at the beginning of the time period. (Any drug for which the first prescription occurs within the first 90 days of the study period is included here.)

**MaxDrugs** The maximum number of drugs the patient was on at one time.

**AverageDrugs** The average number of drugs the patient was on at a time

**UniqueDrugs** The number of distinct drugs that the patient was one over the course of the study period.

**DrugGapNone** The fraction of refills which occurred immediately after the previous prescription ran out (i.e. there was no gap before the refill).

**DrugGapSmall** The fraction of refills which were preceded by a small gap (between 1 and 30 days) after the previous prescription ran out.

**DrugGapMedium** The fraction of refills which were preceded by a medium gap (between 31 and 90 days) after the previous prescription ran out.

**DrugGapLarge** The fraction of refills which were preceded by a large gap (more than 90 days). These likely aren’t refills at all but indicate that the patient went off of the medication for a while.

We included three versions of each prescription variable: one version applies to all drugs, one applies only to chronic drugs, and one only to acute drugs. The versions applying to chronic drugs are prefixed “Chronic” and those applying to acute drugs are prefixed “Acute.”

## References

[CR1] (2006). National healthcare quality report, 2006.

[CR2] American Diabetes Association (2007). Standards of medical care in diabetes–2007. Diabetes Care.

[CR3] Asch SM, McGlynn EA, Hogan MM, Hayward RA, Shekelle P, Rubenstein L, Keesey J, Adams J, Kerr EA (2004). Comparison of quality of care for patients in the veterans health administration and patients in a national sample. Ann Intern Med.

[CR4] Berwick DM (2002). A user’s manual for the iom’s ’quality chasm’ report. Health Aff.

[CR5] Brook EAMRH, Cleary PD (1996). Measuring quality of care - part 2. N Engl J Med.

[CR6] Brook RH, McGlynn EA, Shekelle PG (2000). Defining and measuring quality of care: a perspective from us researchers. Int J Qual Health Care.

[CR7] (2011). National diabetes fact sheet: general information and national estimates on diabetes in the United States, 2011.

[CR8] Corrigan JM (2001). Crossing the Quality Chasm.

[CR9] Davis K, Schoen C, Schoenbaum SC, Doty MM, Holmgren AL, Kriss JL, Shea KK (2007). Mirror, mirror on the wall: An international update on the comparative performance of american health care.

[CR10] Gandhi TK, Cook EF, Puopolo AL, Burstin HR, Haas JS, Brennan TA (2002). Inconsistent report cards: assessing the comparability of various measures of the quality of ambulatory care. Medical Care.

[CR11] Hanchate A, Stukel T, Birkmeyer J, Ash A (2010). Surgery volume, quality of care and operative mortality in coronary artery bypass graft surgery: a re-examination using fixed-effects regression. Health Services and Outcomes Research Methodology.

[CR12] Hastie T, Tibshirani R, Friedman JH (2001). The Elements of Statistical Learning: Data Mining, Inference, and Prediction.

[CR13] Hayward RA (2007). Performance measurement in search of a path. N Engl J Med.

[CR14] Heisler M, Wagner EH (2004). Improving diabetes treatment quality in managed care organizations: Some progress, many challenges. Am J Manag Care.

[CR15] Hofer TP, Hayward RA, Greenfield S, Wagner EH, Kaplan SH, Manning WG (1999). The unreliability of individual physician “report cards” for assessing the costs and quality of care of a chronic disease. JAMA.

[CR16] Kashner TM (1998). Agreement between administrative files and written medical records. a case of the department of veterans affairs. Medical Care.

[CR17] Kerr EA, Krein SL, Vijan S, Hofer TP, Hayward RA (2001). Avoiding pitfalls in chronic disease quality measurement: a case for the next generation of technical quality measures. Am J Manag Care.

[CR18] Leatherman ST, McCarthy D (2002). Quality of Health Care in the United States.

[CR19] Li Y, Dick A, Glance L, Cai X, Mukamel D (2007). Misspecification issues in risk adjustment and construction of outcome-based quality indicators. Health Services and Outcomes Research Methodology.

[CR20] Pham HH, Schrag D, O’Malley AS, Wu B, Bach PB (2007). Care patterns in medicare and their implications for pay for performance. N Engl J Med.

[CR21] Romano PS, Mutter R (2004). The evolving science of quality measurement for hospitals: implications for studies of competition and consolidation. Int J Health Care Finance Econ.

[CR22] Schoenbaum SC, McCarthy D, Schoen C (2007). The Agency for Healthcare Research and Quality's 2006 National Healthcare Quality Report.

[CR23] Scholle SH, Roski J, Dunn DL, Adams JL, Dugan DP, Pawlson LG, Kerr EA (2009). Availability of data for measuring physician quality performance. Am J Manag Care.

[CR24] Schuster MA, McGlynn EA, Brook RH (1998). How good is the quality of health care in the united states?. Milbank Q.

[CR25] Shwartz M, Burgess J, Berlowitz D (2009). Benefit-of-the-doubt approaches for calculating a composite measure of quality. Health Services and Outcomes Research Methodology.

[CR26] Solberg LI, Asche SE, Pawlson LG, Scholle SH, Shih SC (2008). Practice systems are associated with high-quality care for diabetes. Am J Manag Care.

[CR27] (2012). Hospital compare.

[CR28] Weiner JP, Parente ST, Garnick DW, Fowles J, Lawthers AG, Palmer RH (1995). Variation in office-based quality. a claims-based profile of care provided to medicare patients with diabetes. JAMA.

[CR29] Wennberg JE, Roos N, Sola L, Schori A, Jaffe R (1987). Use of claims data systems to evaluate health care outcomes. mortality and reoperation following prostatectomy. JAMA.

[CR30] Wholey D, Christianson J, Finch M, Knutson D, Rockwood T, Warrick L, Team PEHPR (2003). Evaluating health plan quality 1: a conceptual model. Am J Manag Care Spec No.

[CR31] Wholey D, Christianson J, Finch M, Knutson D, Rockwood T, Warrick L, Team PEHPR (2003). Evaluating health plan quality 2: survey design principles for measuring health plan quality. Am J Manag Care Spec No.

